# Overexpression of mGlu_7B_ in Mice: Implications for Neurodevelopmental Disorders

**DOI:** 10.1007/s12035-025-05183-y

**Published:** 2025-07-10

**Authors:** Geanne A. Freitas, Kelly Weiss, Vaishnavi Bavadekar, Sheryl Anne D. Vermudez, Nicole M. Fisher, Aditi Buch, Shalini Dogra, Zixiu Xiang, Rocco G. Gogliotti, Colleen M. Niswender

**Affiliations:** 1 Department of Pharmacology and Warren Center for Neuroscience Drug Discovery, Vanderbilt University, Nashville, TN 37232 USA; 2 Department of Molecular Pharmacology and Neuroscience, Loyola University Chicago, Chicago, IL 60660 USA; 3 Vanderbilt Kennedy Center, Vanderbilt University Medical Center, Nashville, TN 37232 USA; 4 Vanderbilt Institute of Chemical Biology, Vanderbilt University, Nashville, TN 37232 USA; 5 Vanderbilt Brain Institute, Vanderbilt University, Nashville, TN 37232 USA

**Keywords:** Glutamate, Metabotropic, Cognition, Neurodevelopmental disorders, Electrophysiology, Hippocampus

## Abstract

Metabotropic glutamate receptor 7 (mGlu_7_) is a G protein-coupled receptor (GPCR) involved in neurotransmitter release throughout the central nervous system (CNS). Low levels of the receptor are correlated with intellectual disability, autism, repetitive behaviors, and seizures in patients with neurodevelopmental disorders (NDDs), including the disease Rett syndrome. These findings suggest that increasing mGlu_7_ activity may be of therapeutic benefit. In the current manuscript, we report the characterization of a novel transgenic mouse that overexpresses the human *GRM7B* splice variant at approximately fivefold higher levels compared to wild-type (WT) littermates. These animals exhibit a reciprocal decrease in expression of the mouse mGlu_7A_ splice isoform, suggesting feedback regulation of receptor expression. Previous studies from our lab and others have shown that mGlu_7_ activation is permissive for long-term potentiation induction in the hippocampus and amygdala. Here, we identified subtle differences in agonist-modulated hippocampal field recordings in mice overexpressing mGlu_7B_, but no changes in theta burst-induced long-term potentiation. Our lab previously characterized behavioral phenotypes in *Grm7*^*−/−*^ animals that were observed in other animal models of NDDs. Surprisingly, we find here that mGlu_7B_-overexpressing mice exhibit similar phenotypes to previously reported studies in *Grm7*^*−/−*^ animals in repetitive behavior and cognition assays. Overall, these findings suggest that precise control of mGlu_7_ may be required to avoid abnormal phenotypes.

**Supplementary Information:**

The online version contains supplementary material available at 10.1007/s12035-025-05183-y.

## Introduction

Metabotropic glutamate receptor 7 (mGlu_7_) is a group III metabotropic glutamate receptor that regulates presynaptic neurotransmitter release throughout the central nervous system (CNS). Three members of this family, mGlu_4_, mGlu_7_, and mGlu_8_, regulate presynaptic neurotransmitter release widely throughout the CNS [1]. However, in contrast to mGlu_4_ and mGlu_8_, mGlu_7_ only responds to very high levels of glutamate, on the order of high μM to mM. mGlu_7_ is also specifically localized to the presynaptic active zones of glutamatergic and GABAergic synapses, while mGlu_4_ and mGlu_8_ are expressed peri-synaptically [2–4].

Polymorphisms in the *GRM7* gene, which codes for the mGlu_7_ protein, have been linked to complex diseases such as schizophrenia, bipolar disorder, autism, and ADHD [5–20]. Exome sequencing in patients diagnosed with neurodevelopmental disorders (NDDs) has also identified primary missense and nonsense mutations within the *GRM7* gene; these individuals present with severe neurological symptoms that include seizures, developmental delay, ADHD, microcephaly, leukodystrophy, hypomyelination, and intellectual impairments [21–27]. Pathogenic mutations that have been identified in *GRM7* have been characterized as loss-of-function and induce impairments in receptor trafficking and neuronal maturation [26, 28].

In mice, disruptions in mGlu_7_ function are associated with aberrant neuronal activity, contributing to seizures, repetitive behaviors, cognitive impairments, and synaptic plasticity changes [27, 29–38]. mGlu_7_ activation is permissive for the induction of long-term potentiation (LTP) in the amygdala [39] and hippocampus by reducing GABA release [40]. We have also reported blunted motor and EEG responses to the stimulant amphetamine in *Grm7*^*−/−*^ mice [31]. Collectively, these data suggest that mutations or deletions of mGlu_7_ impact cognition, seizures, motor activity, and responses to stimulants [8, 10, 11, 20].

To provide further support for mGlu_7_ as a target in NDDs, we have shown that mGlu_7_ protein levels are significantly decreased in patients with the neurodevelopmental disorder, Rett syndrome (RTT) [41]. RTT is caused by mutations in the methyl CpG binding protein 2 (*MECP2*) gene [42], which encodes an X-linked transcription factor. RTT patients develop seizures, intellectual disability, breathing abnormalities, repetitive behaviors, and autistic features [43–45]. Decreased mGlu_7_ expression is also observed in mouse models of RTT [41, 46], and we have shown that several phenotypes in *Mecp2* heterozygous female (*Mecp2*^+*/−*^) and hemizygous male (*Mecp2*^*−/y*^) mice are corrected after administration of a small molecule positive allosteric modulator (PAM) with mGlu_7_ activity [41]. Overexpression of mGlu_7_ using a viral transgene in the cortex in mouse models of autistic features also improves social interaction, anxiety, and repetitive behavior phenotypes [47]. Additionally, Girard et al. have shown that activation of mGlu_7_ prevents kindling of epileptic seizures in mice and Tassin et al. demonstrated that an mGlu_7_ negative allosteric modulator (NAM) causes sedation and seizures [33, 34]. Together, these findings suggest that low levels of mGlu_7_ may contribute to phenotypes seen in patients with NDDs and provide rationale for increasing mGlu_7_ activity as a potential treatment for NDDs [27, 32, 48].

**Fig. 1:**
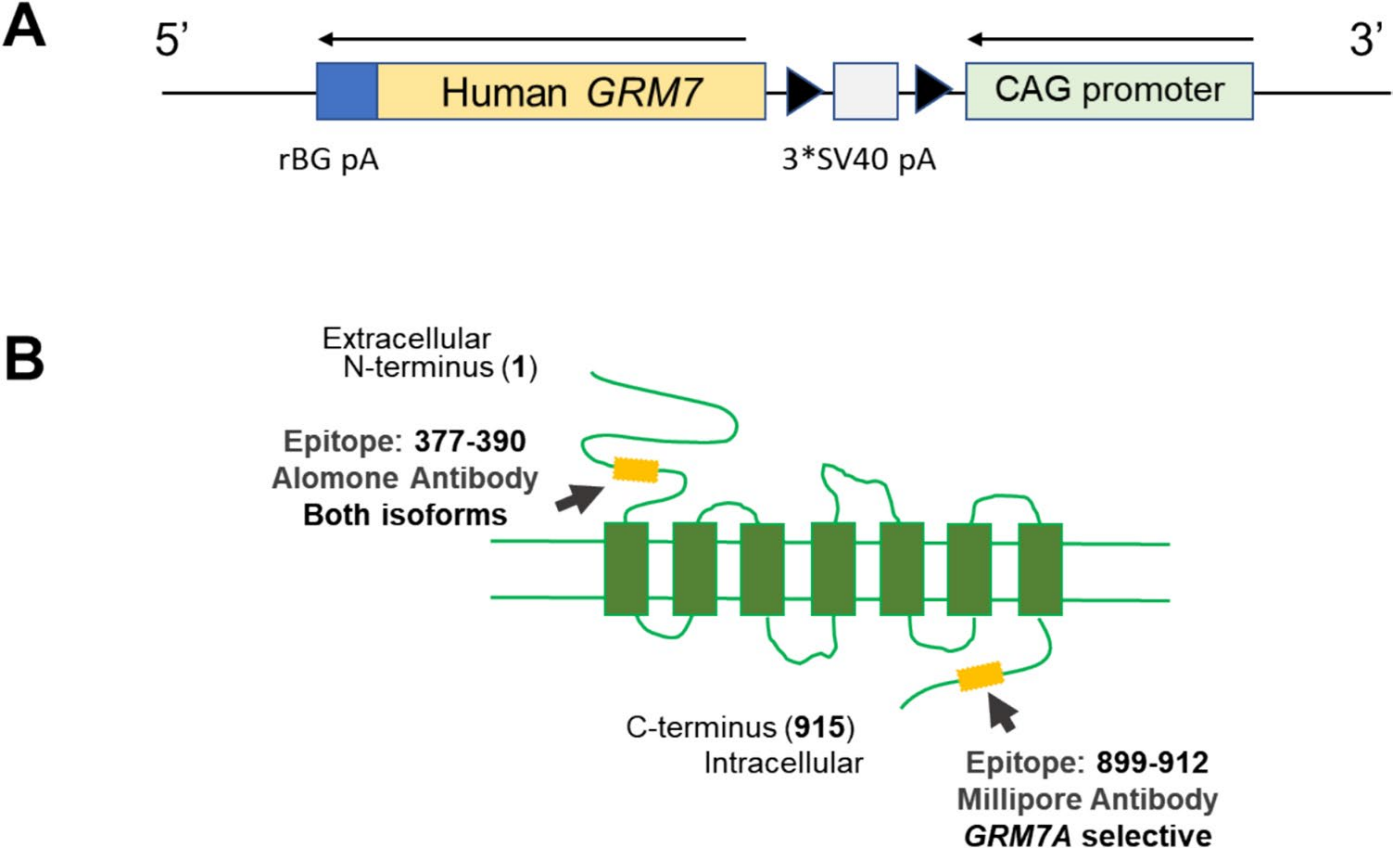
Design of *GRM7B* overexpression allele and localization of antibodies. **A** Targeted allele designed by Cyagen. **B** Schematic of an mGlu_7_ protein protomer with the positions of the epitopes of antibodies. The Alomone anti-mGlu_7_ antibody is N-terminal (recognizes amino acids 377–390) and detects both splice variants, whereas the Millipore antibody is C-terminal (recognizes amino acids 899–912) and differentiates the A and B splice variants of mGlu_7_. Validation of these antibodies using *Grm7*^*−/*^.^*−*^ animals is shown in Supplemental Fig. 2 (Alomone) and [41] (Millipore)

An emerging theme in NDD research is that both loss of function and overexpression of a causative gene, such as fragile X mental messenger ribonucleoprotein 1 (*FMR1*), cyclin-dependent kinase-like 5 (*CDKL5*), or transcription factor 4 (*TCF4*), often cause deleterious phenotypes [49–52]. Another example of this is MeCP2 itself, in which decreases in protein expression or function result in RTT, while increases result in another severe NDD termed *MECP2* duplication syndrome (MDS [53–55]). To further explore *GRM7*’s role as a causative gene for NDDs and to determine consequences of increases in mGlu_7_ activity, we created transgenic mice that overexpress a floxed human *GRM7* transgene via a CRISPR/Cas9 process (Fig. 1). There are two major splice variants of mGlu_7_, A and B [56], and here, we report animals that overexpress the *GRM7B* splice variant. We crossed this line with animals expressing Cre recombinase under the control of the cytomegalovirus (CMV) promoter to cause germline overexpression of the mGlu_7B_ receptor in all tissues, resulting in approximately fivefold increases in receptor mRNA and protein. Here, we report the characterization of expression, effects on synaptic activity and plasticity, and behavioral consequences of mGlu_7B_ overexpression.

## Materials and Methods

### Animals

All animals used in the present study were group housed with food and water given ad libitum and maintained on a 12-h light/dark cycle. Animals were cared for in accordance with the National Institutes of Health Guide for the Care and Use of Laboratory Animals. All studies were approved by the Vanderbilt Institutional Animal Care and Use Committee and took place during the light phase. *GRM7B*-conditional ON (cON) floxed transgenic mice were generated at Cyagen US Inc., and then backcrossed to WT C57BL/6 J mice (The Jackson Laboratory, stock no. 000664) for five generations. The human *GRM7B* gene sequence (accession number NM_181874.2) was cloned as a CAG-loxP-Stop-loxP-human *GRM7* cDNA-polyA cassette (Fig. 1A) into intron 1 of the *Rosa26* locus. Once on a C57BL/6 J genetic background, mice expressing the *GRM7B*-floxed cON allele were crossed with a cytomegalovirus (CMV)-Cre mouse line (The Jackson Laboratory, stock no. 006054 (CMV-Cre)) to achieve recombination in the germline and to pass the recombined allele to progeny*.* Animals with the recombined allele in germ cells were then bred to WT C57BL/6 J mice to remove the Cre allele for experimental evaluation; these animals were termed “*ON/* + ” to reflect one transgenic allele and one wild-type allele. *Grm7*^*−/−*^ knockout mice were cryorecovered from the Mutant Mouse Regional Resource Center (B6.129P2-Grm7^Tm1Dgen^/Mmnc) and used as controls for RNAscope and Western blotting.

### RNAscope In Situ Hybridization Assay

Mice were anesthetized with 5% isoflurane and intracardially perfused with ice-cold sterile saline (0.9%NaCl; KD Medical Catalog, no. RGC3290) followed by perfusion with 4% paraformaldehyde (PFA; Sigma Aldrich Catalog, no.158127). After perfusion, whole brains were quickly removed and post-fixed in 4% PFA overnight and transferred into a 30% sucrose solution. Post-fixed brains were rapidly frozen on crushed dry ice using O.C.T. Compound (Tissue-Plus, Fisher Healthcare). Coronal Sects. (16 µm) of cortex, hippocampus, and amygdala were cut on a cryostat, mounted onto Superfrost™ Plus Microscope Slides (Fisherbrand™, Cat no. 22–037-246), and stored at *−*80 °C. RNAscope assays were performed following the manufacturer’s protocols (Advance Cell Diagnostics (ACD), Hayward, CA, USA). Mounted tissue sections were serially dehydrated in 50%, 70%, 100%, and 100% ethanol for 5 min each at room temperature. Between all pretreatment steps, tissue sections were briefly washed with ultra-pure water. Hydrogen peroxide reagent was applied for 10 min at room temperature. Mounted slices were treated with Protease III for 10 min at 40 °C. Human and mouse *GRM7/Grm7* RNAscope® probes were purchased from ACD-Bio (Human: Cat no. 592871, accession number: NM_ 181,874.2; Mouse: Cat no. 487841-C3, Accession number: NM_177328.3). Amplification steps were performed according to manufacturer’s directions at 40 °C. In between each amplification, sections were washed with 1 × wash buffer. Detection was performed using an OpalDye 570 (Akoya Biosciences Cat no. SKU FP1488001KT) diluted in TSA Buffer (ACD-bio; Cat no. 322810) at 1:500. Sections were incubated for 30 min at 40 °C and rinsed with 1 × wash buffer and incubated with 4′,6-diamidino-2-phenylindole (DAPI, ACD-Bio) to counterstain nuclei. Images were acquired using a confocal microscope (LSM 800/Ryman, Zeiss) with × 20 or × 63 objectives in the inverted configuration at the Vanderbilt Cell Imaging Shared Resource (CISR) Core. The data documentation and analysis were conducted using FIJI ImageJ software.

### Staining Quantification

The amount of *GRM7* was quantified in the cortex, hippocampus, and amygdala from *ON/* + transgenic mice using × 63 RNAscope images stained with *GRM7* probes and obtained using a confocal microscope (LSM 800/Ryman, Zeiss), as specified above. The fluorescence intensity was normalized by the negative control (absence of probes) using ImageJ software. Data were then normalized by subtracting signals determined using negative control images and the fluorescence intensity signal that exceeded the negative control fluorescence signal was calculated. The results were represented as the total mean fluorescence intensity signal, in relative fluorescence units (RFU).

### Quantitative RT-PCR

Total RNA was prepared from ~ 100 mg of hippocampal samples using standard Trizol-chloroform methodology with DNase treatment (Qiagen). cDNA was synthesized from 2 µg total RNA with the SuperScript VILO kit (Thermo Fisher, Waltham, MA). qRT-PCR (CFX96, Bio-Rad, equipment located at the Vanderbilt University Medical Center Molecular Cellular Biology Resource (MCBR) Core) was performed on 50 ng/9 µL cDNA from the hippocampal samples and run in duplicate using PowerUp™ SYBR™ Green Master Mix (ThermoFisher cat no. A25742) with the following primers (5′ to 3′): mouse *Grm7*: (exon 1, forward: CTCGACCAGATCAACAGCGA, reverse: CAGGAGCCGTGGATGCATAA, 298 bp), human/mouse *GRM7/Grm7* (exon 1, forward: GATGAAGTTCCCCTGCTGCG, reverse: CTGGGACCCTTGGCGTG, 150 bp), mouse *Grm4* (forward: CTCCAGCCGCACGCTTGACA, reverse: GTAGGCCGAGTCCTGGA, 157 bp), mouse *Grm8* (forward: CGGAATCTGAACTTGCTCGG, reverse: AAATCCCTAAAGCCTTCCCCC, 227 bp), and *Gapdh* (exon 6, forward: CGACTTCAACAGCAACTCCC, reverse: GCCGTATTCATTGTCATACCAGG, 106 bp). All primer sequences were designed using Primer3 and constructed by Sigma through the Vanderbilt University Medical Center Molecular Cell Biology Resource core. Ct values for each sample were normalized to *Gapdh* expression and analyzed using the delta − delta Ct method [57]. Values exceeding two times the standard deviation were classified as outliers. Each value was compared to the average delta-Ct value acquired for wild-type controls and calculated as percent-relative to the average control delta-Ct for wild type.

### Western Blotting

For total protein extraction, male and female hippocampal tissue samples were homogenized using a hand-held motorized mortar and pestle in radioimmunoprecipitation assay buffer (RIPA) containing 10 mM Tris–HCl, 150 mM NaCl, 1 mM ethylenediaminetetraacetic acid (EDTA), 0.1% sodium dodecyl sulfate (SDS), 1% Triton X-100, and 1% deoxycholate (Sigma). After homogenization, samples were centrifuged at 12,000 *g* for 20 min at 4 °C and the supernatant collected. Protein concentration was determined using a bicinchoninic acid (BCA) protein assay (Pierce, ThermoFisher). Fifty micrograms of total protein from the hippocampus was electrophoretically separated using a 4–20% SDS polyacrylamide gel and transferred onto a nitrocellulose membrane (Criterion™ Blotter, Bio-Rad). Membranes were blocked in Tris buffered saline Odyssey blocking buffer (LI-COR, Lincoln, NE, USA) for 1 h at room temperature. Membranes were probed with primary antibodies overnight at 4 °C: rabbit anti-mGlu_7_ (1:1000, Alomone Labs cat no. AGC-017, recognizes amino acids 377–390 and detects both mGlu_7A_ and mGlu_7B_, Fig. 1B), rabbit anti-mGlu_7_ (1:1000, Millipore/Upstate cat no. 07–239, recognizes amino acids 899–912, specific to mGlu_7A_, Fig. 1B), and mouse anti-tubulin (1:5000, Abcam cat. no. ab44928), followed by fluorescent secondary antibodies: goat anti-rabbit (800 nm, 1:5000, LI-COR) and goat anti-mouse (680 nm, 1:10,000, LI-COR). Fluorescence was detected using an Odyssey infrared imager (LI-COR, Vanderbilt University Department of Pharmacology Core Facilities) then quantified using Image Studio Lite software (*LI-COR*). Values were normalized to tubulin and compared relative to wild-type controls.

### Electrophysiology

Both male and female mice (6 to 8 weeks old) overexpressing mGlu_7B_ and their littermate wild-type controls were anesthetized using 5% isoflurane. The brain was rapidly extracted, blocked and mounted onto a vibratome cutting stage submerged in an ice-cold, oxygenated (95% O_2_/5% CO_2_) N-methyl-d-glucamine (NMDG) solution composed of (in mM): 93 NMDG, 30 NaHCO_3_, 25 glucose, 20 HEPES, 10 MgSO_4_, 5 sodium ascorbate, 3 sodium pyruvate, 2.5 KCl, 2 thiourea, 1.2 NaH_2_PO_4_, and 0.5 CaCl_2_ titrated with HCl to a pH between 7.3 and 7.4 with an osmolality of approximately 305 to 310 milliosmoles (mOsm). Coronal slices containing the hippocampus (400 µm) were hemisected, then transferred to NMDG solution kept at 32 °C for 10 min for recovery. Slices were then transferred to a holding chamber containing artificial cerebral spinal fluid (aCSF) solution composed of (in mM) the following: 126 NaCl, 25 NaHCO_3_, 11 glucose, 2.5 KCl, 2.5 CaCl_2_, 1.2 NaH_2_PO_4_, 1 MgSO_4_ supplemented with 0.4 mM ascorbic acid with an osmolality adjusted to 293–297 mOsm at room temperature for 60–90 min before recording (fEPSPs). Slices containing the hippocampus were transferred to a submerged recording chamber (Warner Instruments, CT, USA) perfused with oxygenated aCSF maintained at 32 °C using an in-line heater (Warner Instruments, CT, USA) at a rate of 2 mL/min. A concentric bipolar stimulating electrode (FSC Inc.) was placed near the boundary of the SC-CA1 synapse to deliver the electrical stimulation (200-μs duration), and a glass recording pipette of resistance 2–3 MΩ filled with aCSF was placed in the stratum radiatum region to record paired-pulse fEPSPs. Input–output (I/O) curves were generated for each slice and the stimulus intensity for the subsequent experiment was determined as the intensity that yielded 40–50% of the maximal response. The paired pulse ratio (PPR) was calculated by dividing the slope of second response to the first response. PPRs were also acquired using electrical pulses at varying interstimulus intervals (ISI). For long-term potentiation (LTP) studies, paired-pulse fEPSPs were evoked by delivering paired pulses of 200-μs duration with a 50 ms ISI, spaced every 20 s [58, 59]. After stable baseline signals were collected for 10 min, a theta burst stimulation (TBS) protocol consisting of nine bursts of four electrical stimuli applied at 100 Hz, repeated every 230 ms, was applied to generate LTP, and then paired-pulse fEPSPs were recorded for 50 min [58, 59]. The data were acquired and processed using pClamp 10.6 software (Molecular Devices). The slopes of three consecutive sweeps were averaged and were normalized to the average slope during the baseline recordings.

### Behavioral Assays

All behavioral experiments were conducted in male and female mice that were 6 to 10 weeks of age at the Vanderbilt University Medical Center Mouse Neurobehavioral Lab Core. Mice were utilized in multiple assays, conducted in the following order: open field, elevated zero maze, clasping, and contextual fear conditioning, with a minimum of 5 days elapsed between each assay. For each assay, mice were habituated to the testing room for at least 30 min prior to the experiment. Quantification was performed either by a researcher blinded to the genotype or by automated software.

### Open Field

Mice were placed in the activity chamber for 60 min. Exploratory and locomotor activities were quantified using Activity Monitor software by assessing beam breaks in the X, Y, and Z axes (Med Associates Inc.).

### Elevated Zero Maze

Mice were placed on an elevated zero maze, where two regions were closed (contain walls) and two regions were open (no walls). Mice were allowed to freely explore the maze for 5 min under full light conditions (~ 700 lx in the open regions, ~ 400 lx in the closed regions), and the percent time spent in the open versus closed regions and number of region-transitions were measured. The assay was tracked by a mounted camera and analyzed by automated AnyMaze software (Stoelting, Wood Dale, IL, USA).

### Hindlimb Clasping

Hindlimb clasping was assessed as described in [41, 60] by suspending a mouse by its tail and video recording clasping dynamics for a period of 1 min, and clasping was scored by a blinded reviewer. Recording occurred 3 days in a row, and the average time spent clasping was documented. Clasping was defined as the number of seconds spent clasping with one or more hind paws.

### Contextual Fear Conditioning

On day 1 (training day), mice were placed into an operant chamber for a 3-min acclimation period to record baseline behavior; immediately following, training was performed by delivering two foot shocks (lasting 2 s each at an intensity of 0.7 mA, 60-s intertrial interval) via a grid floor (Med Associates, Inc., St. Albans, VT, USA) in the presence of a 10% vanilla odor cue. Mice remained in the context for an additional 30 s after the second foot shock. On day 2 (testing day), 24 h after conditioning, mice were placed back into the operant chamber for 3 min in the presence of a 10% vanilla odor cue. No foot shocks were delivered. Percentage of time spent freezing was measured by Video Freeze software (Med Associates Inc., St. Albans, VT, USA).

### Amphetamine-Induced Hyperlocomotion

Amphetamine-induced locomotion was performed as previously described [61–69]. Mice were acclimated to an open field chamber for 30 min and then administered amphetamine (2.25 mg/kg, *s.c.*), and changes in locomotor activity were monitored in an open field for an additional 60 min.

### Statistical Analyses

Statistics were carried out using GraphPad Prism and Excel (Microsoft). All data shown represent mean ± SEM. Statistical significance between genotypes was determined using Student’s *t* tests or ordinary way ANOVA with post hoc tests. Sample size (denoted as *N*), statistical test, and results of statistical analyses are specified in each figure legend with *p* values listed in either the text or figure legend.

## Results

To assess the consequences of overexpression of mGlu_7_, a transgene encoding the human mGlu_7B_ receptor was inserted into the *Rosa26* locus on mouse chromosome 6 under control of a CAG promoter with a floxed stop cassette (3*SV40 pA) sequence to render the allele conditional-on (c-ON, Fig. 1A). These mice were then crossed to animals expressing Cre recombinase under the control of the cytomegalovirus promoter (CMV-Cre, B6.C-Tg(CMV-cre)1Cgn/J, Jackson Labs) to cause germline removal of the stop cassette and expression in all tissues; mice with the *GRM7B* recombined allele were then further bred to C57/Bl6J animals to remove the CMV-Cre allele. Test mice were given the nomenclature “*ON/* + ” to denote heterozygosity of a transgenic *GRM7* allele and one wild-type (WT) allele. mGlu_7_ is alternatively spliced to generate two major isoforms: mGlu_7A_ and mGlu_7B_ that differ in their C-termini [56]. In this study, we overexpressed *GRM7B* due to the availability of antibodies that selectively recognize the mGlu_7A_ splice variant (the “Materials and Methods” section), allowing the detection of effects on the mouse mGlu_7A_ receptor in the context of overexpression of mGlu_7B_. Shown in Fig. 1B is a schematic of an mGlu_7_ monomer with the positions noted that correspond to the recognition sequences of the various antibodies employed in our characterization studies. The anti-mGlu_7_ Alomone antibody (AGC-017) recognizes an N-terminal sequence from amino acids 377–390 and interacts with both the A and B splice variants. The anti-mGlu_7_ Millipore antibody (07–239) recognizes an epitope from amino acids 899–912; these amino acids are specific to the A splice variant.

**Fig. 2:**
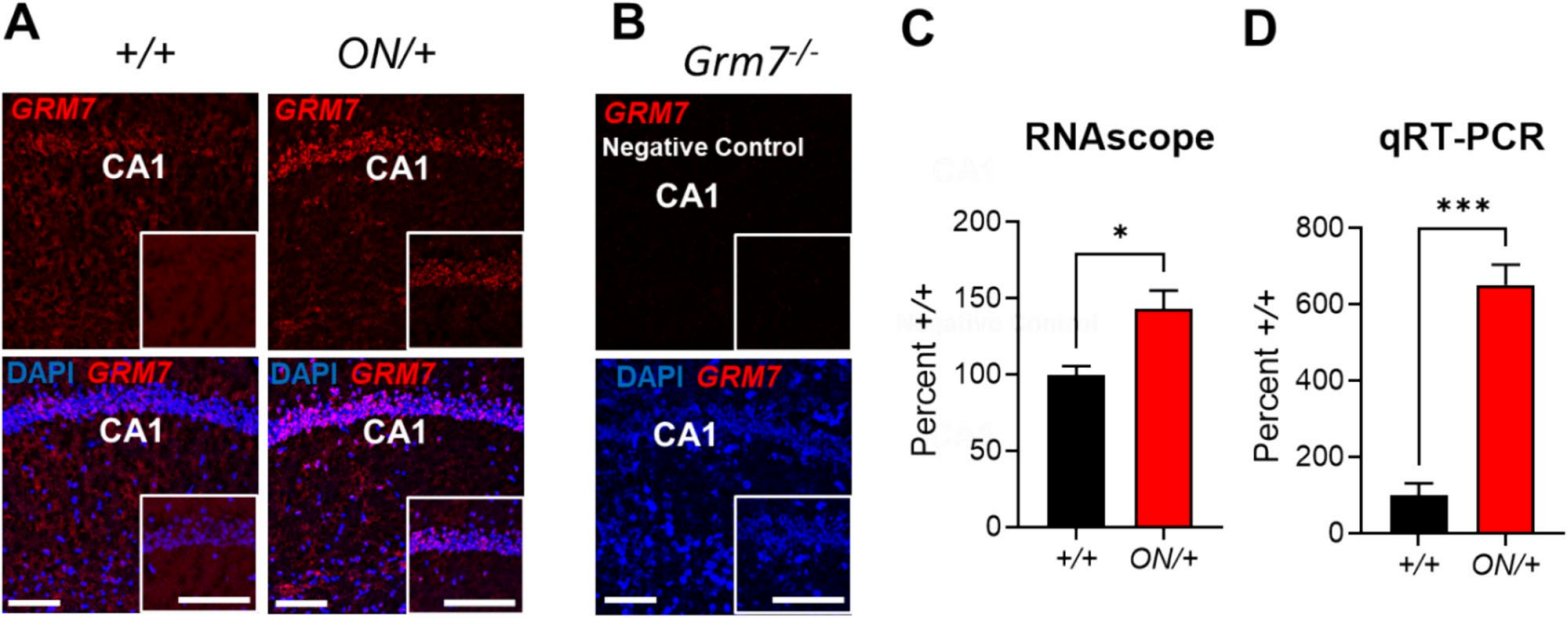
*GRM7B* mRNA is significantly increased in *ON/+* hippocampal tissue compared to +/+ tissue as assessed by RNAscope and quantitative RT-PCR. **A **RNAscope staining in the CA1-hippocampus of *ON/+ *showed a visual increase of *GRM7* expression in the CA1 region compared to +/+ mice. **B **Control experiments in *Grm7 KOs* showed no staining. Inset images point to the signal differences detected between groups. Red: *GRM7* mRNA; Blue: nuclei counterstain with DAPI. Scale bar: 100 µm. **C** Bar graphs showing the difference in the total mean fluorescence intensity signal normalized to the *+/+* signal in hippocampus of *+/+ *(black) versus *ON/+* (red) mice (*+/+*, 100.0 ± 5.6%, *ON/+*, 142.8 ± 12.2 %, n=4-5 mice per group, *p=0.037 by unpaired t-test. **D ***GRM7* mRNA expression was also increased in the hippocampus of* ON/+ *(red) versus *+/+* (black) mice as assessed using quantitative RT-PCR. *+/+*, 100.0 ± 31.2, *p=0.037; *ON/+*, 651.2 ± 53.2%, n=4 per group, ***p=0.0001. *GRM7*: Metabotropic Glutamate Receptor 7; CA1: Cornu Ammonis 1

We evaluated the expression of mGlu_7_ at both the mRNA and protein levels in WT (+ */* +) and *ON/* + mouse lines using RNAscope, quantitative RT-PCR (qRT-PCR), and Western blotting. As shown in Fig. 2A, an increase in expression of mRNA for *GRM7* was detected using RNAscope in the hippocampus of *ON/* + mice compared to + */* + control mice. In Fig. 2B, negative control staining in *Grm7*^−/−^ slices shows specificity of the RNAscope probe for *GRM7/Grm7*. In Fig. 2C, quantitation of the RNAscope staining in the hippocampus between + */* + and *ON/* + mice revealed a significant increase in the *ON/* + animals (*p = 0.037, unpaired *t* test). In Fig. 2D, these results were confirmed from hippocampal tissue punches by quantitative RT-PCR (***p = 0.0001, unpaired *t* test); these differences in expression between +/+ and *ON/* + in the two mRNA detection techniques may be due to differences in tissue capture. For example, in RNAscope, just the CA1 region was quantified; in qRT-PCR, the whole hippocampus was evaluated. We also quantified expression in the cortex and amygdala using RNAscope and observed significant increases in *GRM7* mRNA levels in both regions (*p = 0.033 for cortex and *p = 0.026 for amygdala, unpaired *t* tests, SI Fig. 1). Additionally, we used mouse-specific primers in the hippocampus for qRT-PCR, and mGlu_7_ mRNA levels of the mouse transcript were not statistically different between genotypes (+/+, 100.0 ± 5.7%; ON/+, 139.3 ± 23.5, mean ± SEM, *n* = 3–4, *p* = 0.113, unpaired *t* test). We also tested the levels of mouse mGlu_4_ and mGlu_8_, the other widely expressed group III mGlu receptors. These experiments revealed no significant differences between genotypes for either mGlu_4_ (+/+, 100.0 ± 11.7%; ON/+, 133.3 ± 24.6%, mean ± SEM, *n* = 4, *p* = 0.267, unpaired *t* test) or mGlu_8_ (+/+, 100.0 ± 11.8%; ON/+, 105.7 ± 7.7%, mean ± SEM, *n* = 3–4, *p* = 0.738, unpaired *t* test).

**Fig. 3:**
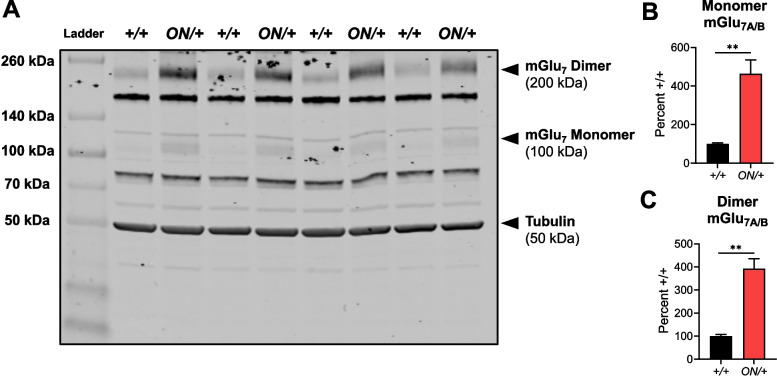
mGlu_7_ protein expression is increased in the hippocampus of *ON/* + mice compared to + */* + mice. **A** Western blot using the Alomone antibody shows increases in the expression of monomeric (~ 100 kD, **B**) and dimeric (~ 200 kD, **C**) forms of mGlu_7A_ and mGlu_7B_ proteins in *ON/* + mice compared to + */* + animals. *Grm*_*7*_^−/−^ mice were used as negative control for this antibody (SI Fig. 2). Monomer expression: 100.0 ± 5.5%, + */* +, versus 464.2 ± 71.7%, *ON/* +, *n* = 3–4 per group, **p = 0.0078 by unpaired *t* test. Dimer expression: 100.0 ± 7.1%, + */* +, versus 392.0 ± 44.0%, *ON/* +, *n* = 3–4 per group, **p = 0.0026 by unpaired *t* test

**Fig. 4:**
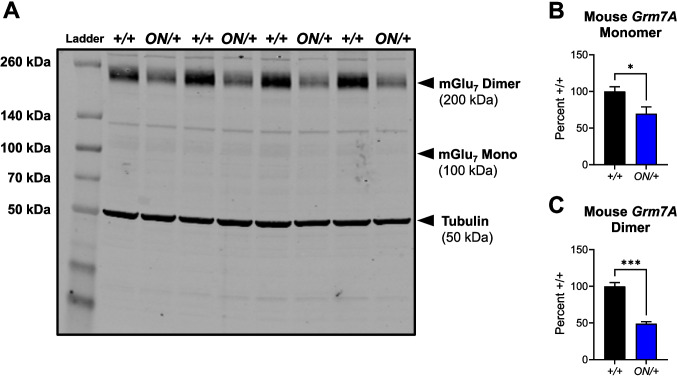
Expression of mouse mGlu_7A_ protein in the hippocampus of *ON/*+. mice is decreased relative to + */* + animals. **A** Using the Millipore anti-mGlu_7A_ antibody, tissues from + */* + or *ON/* + mice were probed for the expression of mGlu_7A_. **B** Monomer (100.0 ± 7.1%, + */* +, versus 69.6 ± 9.4%, *ON/* +, *n* = 4 per group, *p = 0.0368, unpaired *t* test) and **C** dimer (100.0 ± 7.1%, + */* +, versus 49.1 ± 2.6%, *ON/* +, *n* = 4 per group, ***p = 0.001, unpaired *t* test, *n* = 4 animals per genotype)

We next evaluated protein levels of mGlu_7_ using the two distinct antibodies shown in Fig. 1B. When blots were probed with the Alomone antibody that recognizes both splice variants (Fig. 3A; Supplemental Fig. 2 shows lack of detection of mGlu_7_ monomer and dimer in *Grm7*^−*/*−^ mice), hippocampal samples from *ON/* + mice showed significantly greater expression of both the monomeric and dimeric forms of the mGlu_7_ protein when compared to samples from WT controls (monomer expression: **p = 0.0078 by unpaired *t* test, Fig. 3B; dimer expression: **p = 0.0026 by unpaired *t* test, Fig. 3C). As the Millipore antibody is selective for the A splice variant, we also examined the regulation of the mouse mGlu_7A_ protein when *GRM7B* was overexpressed (validation of this antibody using *Grm7*^−*/*−^ controls was performed in [41]). As shown in Fig. 4A, the endogenous mouse mGlu_7A_ monomer (*p = 0.0368, unpaired *t* test, Fig. 4B) and dimer (***p = 0.001, unpaired *t* test, Fig. 4C) proteins were significantly decreased in the hippocampus when *GRM7B* was overexpressed. As we did not detect changes in mGlu_7_ mRNA expression by qRT-PCR, this suggests that there is downregulation of the endogenous protein when mGlu_7_ expression is increased.

**Fig. 5:**
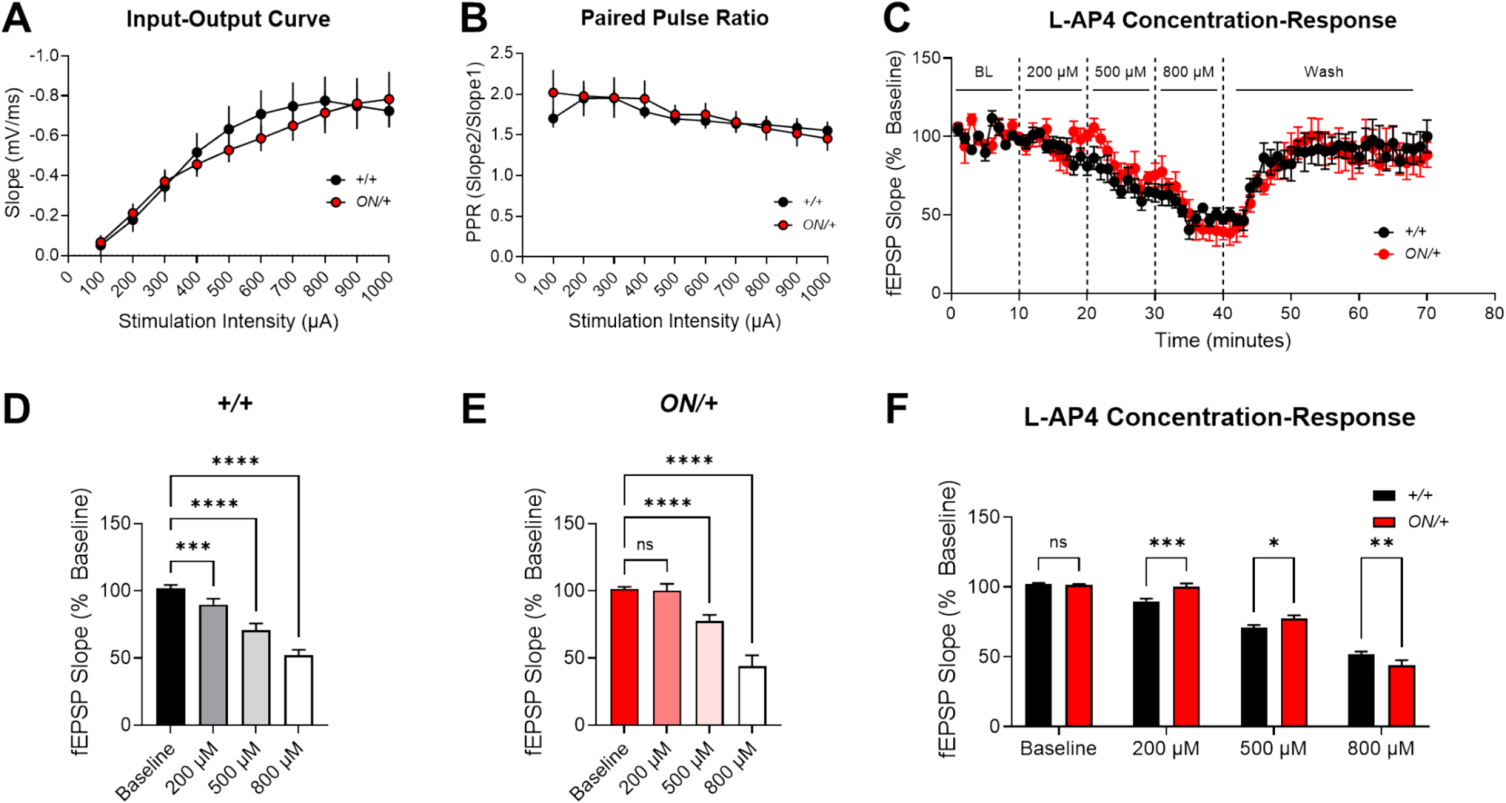
SC-CA1 recordings from *ON/* + mice do not show differences in basal synaptic activity but demonstrate subtle changes in response to the group III mGlu receptor agonist, L-AP4, compared to + */* + mice. Slice prepared from + */* + (black) and *ON/* + (red) mice did not show significant differences in **A** input–output curves or **B** paired pulse ratios across a range of stimulus intensities. **C** Application of increasing concentrations of L-AP4 progressively decreases fEPSPs. **D** These decreases were significant for each L-AP4 concentration versus baseline for +/+ mice. **E** In contrast, the response to a 200 μM concentration of L-AP4 was not significantly different from baseline in *ON/* + mice, suggesting a shift in sensitivity to the right to L-AP4 in *ON/* + mice compared to controls. **F** Responses to L-AP4 were compared for both genotypes, showing significant impairments in *ON/* + slices in blocking the response to 200 (+ */* +, 89.9 ± 1.8, *n* = 6 slices; *ON/* +, 100.2 ± 2.3, *n* = 5 slices; ***p = 0.0009) and 500 μM (+ */* +, 70.8 ± 2.1, *n* = 6 slices; *ON/* + *,* 77.6 ± 2.1, *n* = 5 slices; *p = 0.021) concentrations of L-AP4, and an enhanced response (+ */* +, 52.2 ± 1.6, *n* = 6 slices; *ON/* +, 44.0 ± 3.6, *n* = 5 slices; **p = 0.0065) at the 800 μM concentration

To assess functional effects of *GRM7* overexpression on synaptic transmission, we employed ex vivo electrophysiology in slices of the hippocampus. We have shown that mGlu_7_ activity modulates field excitatory postsynaptic potentials (fEPSPs) and is permissive for the induction of LTP at hippocampal Schaffer collateral-CA1 (SC-CA1) synapses in WT mice by reducing GABA release [40, 70, 71]. *Grm7*^−/−^ mice also exhibit reduced short-term plasticity at these synapses [30]. We first determined input–output curves over a range of stimulus intensities (Fig. 5A). The slope of fEPSPs was not significantly different for the + */* + (black) and *ON/* + (red) genotypes (two-way mixed effects analysis, *p* = 0.829 for genotype; *n* = 24, + */* +; 14, *ON/* +)*.* We also measured paired pulse ratios across a range of stimulation intensities and observed no significant difference between the genotypes (two-way mixed effects analysis, *p* = 0.773 for genotype).

**Fig. 6:**
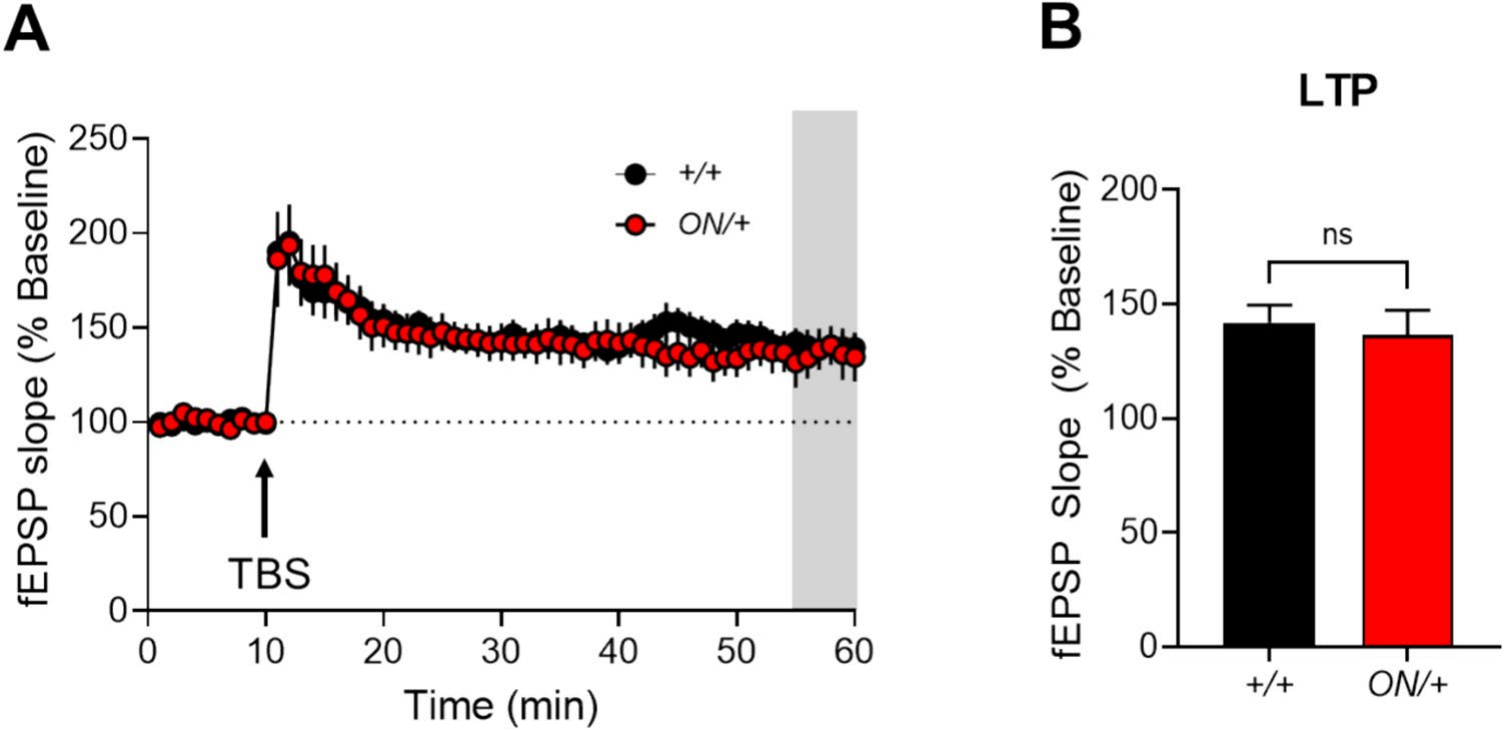
Long-term potentiation (LTP) at SC-CA1 synapses induced by theta burst stimulation is not different between + */* + and *ON/* + genotypes.** A** fEPSP slopes in response to a theta burst stimulation. **B** LTP was measured during the last 5 min of the recording (55–60 min, shaded region, + */* +, black, 142.0 ± 7.6, *n* = 16 slices; *ON/* +, red, 136.4 ± 11.0, *n* = 11 slices; unpaired *t* test, *p* = 0.668)

We have shown that application of mGlu_7_ agonists reduces fEPSPs at SC-CA1 synapses in the hippocampus [40, 70, 71]. For consistency with the agonist used in previous studies and also to account for potential changes in mGlu_8_ activity as it is also expressed at this synapse in neonatal animals [71], we evaluated the ability of the group III mGlu receptor agonist, L-AP4, to modulate fEPSPs by applying increasing concentrations of L-AP4 to slices across a concentration range of 200 to 800 μM (Fig. 5C; black symbols are + */* + slices and red symbols are *ON/* + slices). Relative responses during the L-AP4 addition versus baseline are shown for both + */* + (Fig. 5D, gray/black) and *ON/* + (Fig. 5E, pink/red) slices. These studies showed significant reductions in normalized fEPSP slope from + */* + animals at all three L-AP4 concentrations as compared to the baseline (Fig. 5D). In contrast, *ON/* + mouse slices showed no significant difference from baseline at the 200 µM concentration of L-AP4 but did exhibit significant differences at higher concentrations (Fig. 5E). When the responses to L-AP4 at each concentration were directly compared between genotypes, we observed significant impairments in fEPSP slope depression at the 200 (***p = 0.0009; *N* = 5–6 per genotype) and 500 μM (*p = 0.021) concentrations of L-AP4 and significantly enhanced depression (**p = 0.0065) in *ON/* + slices at the 800 μM concentration (Fig. 5F). Finally, we compared slices from both genotypes for their ability to manifest LTP in response to a theta burst stimulation (TBS) protocol (Fig. 6). These studies showed no significant changes in LTP between genotypes when responses were assessed during the last 5 min (55–60 min) of the recording (*p* = 0.668, unpaired *t* test, Fig. 6).

**Fig. 7:**
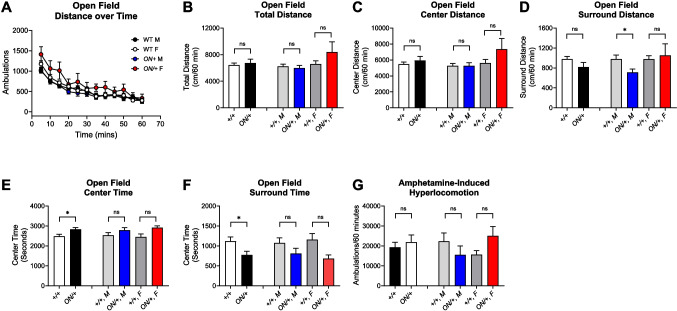
*ON/* + mice do not exhibit gross locomotor changes but exhibit increased time in the center of the open field when sexes are combined. **A** + */* +, 13 males (black) and 17 females (white); and *ON/* +, 13 males (blue) and 6 females (red) were assessed for locomotor responses in an open field. **B** Total distance was not different between genotypes (*p* = 0.6195) and not significantly different between males of each genotype (light gray, *n* = 13, blue, *n* = 13, *p* = 0.6129) or females of each genotype (dark gray, *n* = 17, red, *n* = 6, *p* = 0.1412). **C** Total distance in the center was not different by genotype (*p* = 0.4013) or when separated by sex (males, *p* = 0.9945; females, *p* = 0.1180). **D** Total distance in the surroundings was not significantly different in *ON/* + mice (black) compared to +/+ (white, *p* = 0.1040), but there was a significant reduction in males (*p = 0.0151), but not females (*p* = 0.6790). **E**, **F** Total time in the center was significantly higher and total time in the surroundings was significantly lower in *ON/* + compared to +/+ mice genotype (*p = 0.0240); when separated by sex, values did not reach significance (males, *p* = 0.1701; females, *p* = 0.0943). **G** +/+ (six males, five females) and ON/+ (four males and eight females) were administered amphetamine, and locomotor responses were measured. The locomotor response to amphetamine was not different by genotype (*p* = 0.5663) or when separated by sex (males, *p* = 0.3086; females, *p* = 0.1571). Results are mean ± SEM and all statistical comparisons are unpaired *t* tests between genotypes

We next evaluated + */* + and *ON/* + test mice using a series of behavioral assays that we and others have characterized in *Grm7*^−*/*−^ mice [26, 29, 31, 36–38]. This battery included the following behaviors: spontaneous locomotor activity, anxiety using an elevated zero maze, stereotypic behavior as reflected by hindlimb clasping, and cognition using a contextual fear conditioning paradigm. A separate cohort of mice was also evaluated for the response to amphetamine as we have shown that *Grm7*^−/−^ mice exhibit blunted locomotor responses to amphetamine [31]. In the open field (Fig. 7A, B), we observed no differences in total distance traveled between the + */* + and *ON/* + genotypes when males and females were combined or when they were separated by sex. When distance measurements were separated into distance in the center (covering 80% of the chamber) or the surroundings, we observed no significant difference in the distance travelled in the center when sexes were combined or when evaluated by sex (Fig. 7C); however, we did observe a significant decrease in distance traveled in the surrounding area in male *ON/* + compared to male + */* + mice (Fig. 7D, *p = 0.0151). Total time in the center was increased and time in the surrounding was decreased between genotypes (Fig. 7E, F, *p = 0.0240), but when separated by sex, values did not reach significance. We also assessed the behavioral response to amphetamine, and these studies revealed no differences between + */* + and *ON/* + animals or when separated into males and females (Fig. 7G). This was in contrast to *Grm7*^−*/*−^ mice, which we have previously shown exhibit an abnormally blunted response in amphetamine-induced hyperlocomotion [31].

**Fig. 8:**
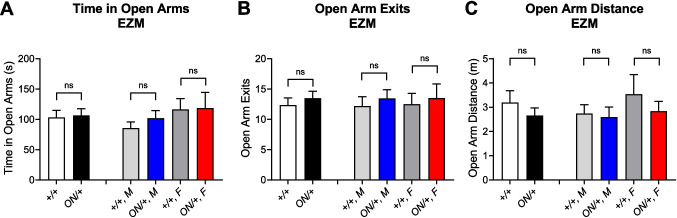
Compared to +/+ mice, ON/+ animals exhibit significant increases in hindlimb clasping and decreases in conditioned fear responses. **A** When assessed for hindlimb clasping, ON/ + mice exhibited a significant increase in clasping time compared to +/+ animals (+/+, 0.6 ± 0.2 s; ON/ +, 9.4 ± 1.5 s, ****p<0.0001, *n*=24 + / +, 19 ON/ +). When separated by sex, there was a significant difference between males (+/+ light gray (*n*=12), and ON/+ blue (*n*=13), ****p< 0.0001) or females (+/+ dark gray (*n*=13), and ON/+ red (*n*=6), **p=0.0022). **B** In a contextual fear conditioning paradigm, ON/ + mice exhibited a significant decrease in freezing time compared to +/+ animals (**p=0.0055, *n*=15 +/+, 14 ON/+ mice). When separated by sex, there was a significant difference between males (+/+ light gray (*n*=7), and ON/+ blue (*n*=10), mean ± SEM, *p=0.0357), but values in females did not reach significance (+/+ dark gray (*n*=8), and ON/+ red (*n*=3), *p*=0.1254). Results are mean ± SEM, and all statistical comparisons are unpaired t tests between genotypes

Increased time in the center area of an open field chamber is often interpreted as anxiolytic. To assess potential effects on anxiety in a more commonly used anxiety task, we used an elevated zero maze test. In this task, *ON/* + mice were not different in terms of time in the open arms of the maze or differences in open arm exits or distance when compared to + */* + animals, and separation of animals into males and females revealed no differences (Fig. 8A–C). Therefore, while the open field results may have indicated a subtle effect on anxiety, we did not observe an anxiolytic effect in a maze-based task.

**Fig. 9:**
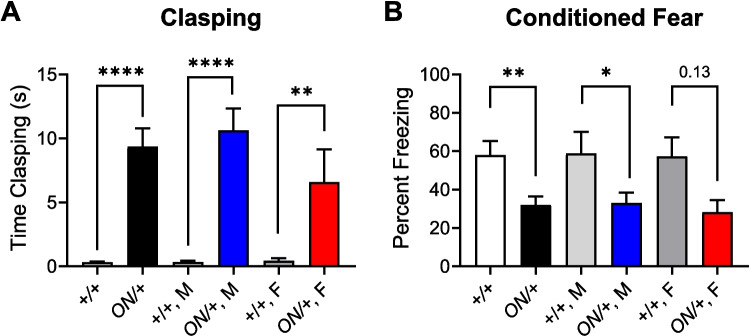
Compared to *+/+* mice, *ON/+* animals exhibit significant increases in hindlimb clasping and decreases in conditioned fear responses. **A** When assessed for hindlimb clasping, *ON/+* mice exhibited a significant increase in clasping time compared to *+/+* animals ((*+/+*, 0.6 ± 0.2 s; *ON/+*, 9.4 ± 1.5 s, ****p<0.0001, *n*=24 *+/+*, 19 *ON/+*)*.* When separated by sex, there was a significant between males (*+/+*, light gray (*n*=12) and *ON/+*, blue (*n*=13), ****p<0.0001) or females (*+/+*, dark gray (*n*=13) and *ON/+*, red (*n*=6), **p=0.0022). **B** In a contextual fear conditioning paradigm, *ON/+* mice exhibited a significant decrease in freezing time compared to *+/+* animals (**p=0.0055, *n*=15 *+/+*, 14 *ON/+* mice). When separated by sex, there was a significant difference between males (*+/+*, light gray (*n*=7) and *ON/+*, blue (*n*=10), mean ± SEM, *p=0.0357), but values in females did not reach significance (*+/+*, dark gray (*n*=8) and *ON/+*, red (*n*=3), *p*=0.1254). Results are mean ± SEM, and all statistical comparisons are unpaired *t* tests between genotypes

We and others have shown that mouse models of RTT, which exhibit significantly reduced mGlu_7_ levels, display a profound clasping phenotype when mice are suspended by the tail [41, 60, 72–75]. *Grm7*^−/−^ and *Grm7* mutant knock-in mice (mice homozygous for an I154T mutation) exhibit a similar clasping phenotype, which can be characterized as a type of repetitive behavior [26, 31]. In our previous knockout studies, we tracked clasping over time and found a significant effect of genotype (****p < 0.0001) between *Grm7*^+*/*+^,* Grm7*^+*/*−^, and *Grm7*^−*/*−^ animals, with significant effects using post hoc tests between *Grm7*^−*/*−^ and *Grm7*^+*/*+^ as well as *Grm7*^−*/*−^ and *Grm7*^+*/*−^ animals of ****p < 0.0001 at all time points (5, 10, 15, and 20 weeks) tested [31]. For I154T animals, we also observed clasping at 8–10 weeks of age [26]. Surprisingly, we also found increases in clasping score in *ON/* + mice when compared to controls and significant clasping levels were observed in both male and female animals (Fig. 9A). We next performed an assessment of associative learning and memory by subjecting mice to a contextual fear conditioning test to measure freezing to a previously aversive stimulus, a task involving the hippocampus. On day 1, mice were trained to associate their environment with a mild foot shock. On day 2, 24 h after shock administration, we evaluated the long-term memory component, and mice were placed back in the same context without foot shock. In this assay, *ON/* + mice exhibited decreased freezing behavior compared to control littermates (Fig. 9B); when separated by sex, males were significantly different but effects in females did not reach statistical significance. Much like hindlimb clasping, we have previously reported impaired associative learning in *Grm7*^−/−^ mice and I154T/154 T mice ([26, 31], *Grm7*^+*/*+^ mice 71.0 ± 3.1% freezing, *Grm7*^+*/*^, 70.3 ± 3.6% freezing, *Grm7*^−*/*−^, 42.4 ± 3.2% freezing).

## Discussion

In the current study, we generated animals that overexpress the mGlu_7B_ splice variant as a tool to explore the consequences of increased expression of the receptor as well as to develop a mechanism to genetically increase mGlu_7_ in the context of mouse models of NDDs. Our findings demonstrate an approximately fivefold increase in mGlu_7_ mRNA and protein expression after recombination of the targeted allele using a CMV-Cre mouse line, which results in germline recombination [76]. The availability of antibodies that differentiate between the isoforms allowed for the quantification of the mouse mGlu_7A_ protein isoform in the context of human *GRM7B* gene overexpression, and our results indicate a reciprocal decrease in mouse mGlu_7A_ expression when human mGlu_7B_ is overexpressed. As we did not observe significant differences in mouse mGlu_7_ mRNA expression between genotypes, this suggests that there is regulation at the post-translational level, and future studies will explore the mechanism for this decrease.

We have previously shown that, during development, hippocampal SC-CA1 synapses express a group III mGlu receptor with pharmacology consistent with the mGlu_8_ receptor [71]. In adult animals, field recording responses to the group III mGlu receptor agonist, L-AP4, “switch” to an mGlu_7_-like phenotype [71, 77]. The conclusion that mGlu_7_ was the primary receptor expressed at SC-CA1 in adult animals was made based on several observations: the increased concentration of L-AP4 required to decrease field EPSPs at SC-CA1 as animals aged which mirrors the low affinity of L-AP4 for mGlu_7_ compared to mGlu_8_; the observation that the effect of an mGlu_8_ agonist, (*S*)-DCPG, in decreasing fEPSPs was lost as the animals aged; control studies demonstrating a lack of mGlu_4_-mediated responses at SC-CA1; and experiments with a new agonist with improved potency at mGlu_7_ compared to mGlu_8_ [40, 71]. We have also reported that knockout of mGlu_7_ leads to decreases in input/output (I/O) responses at high stimulus intensities (> 500 µA) and decreases in PPR but no significant decreases in LTP [31] when compared using ANOVA to *Grm7*^+*/*+^ and *Grm7*^+*/*−^ animals; we hypothesize that this latter observation may be due to compensation by other mGlu receptors. These previous findings suggested that overexpression of mGlu_7_ might change I/O curves or PPR in the opposite direction due to constitutive activity or potentially enhanced glutamate sensitivity in the context of overexpression. As shown in Fig. 5 A and B, however, this does not appear to be the case, as I/O curves and PPR responses were not significantly different across a range of stimulus intensities in + */* + versus *ON/* + animals. While mGlu_7_ has been reported to have high constitutive activity [78], these results appear to more closely align with the hypothesis that mGlu_7_ activity at SC-CA1 synapses requires agonist activation.

When progressively increasing concentrations of L-AP4 were applied to brain slices and fEPSPs were measured, we found small but significant differences in response to L-AP4, with *ON/* + mice showing blunted responses to low L-AP4 concentrations but increased responses to higher concentrations. In vitro, the EC_50_ of L-AP4 is approximately 200 μM at mGlu_7_ [79, 80], and + */* + mice significantly responded to the 200 µM concentration, but *ON/* + mice did not. This suggests a rightward shift in the concentration–response for L-AP4, perhaps due to mGlu_7_ desensitization or internalization. Pelkey et al. have shown that mGlu_7_ is rapidly internalized in neurons after agonist activation [81, 82]. This may also explain the lack of effect of mGlu_7_ overexpression on LTP in *ON/* + mice. mGlu_7_ activation reduces GABAergic synaptic responses in CA1 pyramidal cells, allowing for LTP induction at SC-CA1 synapses [40]. If substantial levels of the overexpressed receptor are rapidly internalized, this may cause a lack of observable effect in LTP; it is also possible that the level of mGlu_7_ in + */* + mice is already sufficient to cause maximal inhibition of GABA release and further increases in receptor expression do not affect this process. Whole-cell patch clamp recordings will be needed to examine this further. Additionally, the line of *ON/* + mice used here overexpresses mGlu_7_ in all cells, and an effect on LTP may be apparent when the receptor is increased in specific cell types, such as GABAergic interneurons. As the allele generated here is floxed, we are now using cell-type–specific Cre lines and viruses to generate tissue-specific increases in *GRM7B* to systematically address the explore the impact of increasing mGlu_7_ levels in select neuronal subtypes to determine effects on LTP.

We previously demonstrated that LTP was not, somewhat surprisingly, significantly decreased in *Grm7*^−/−^ mice compared to *Grm7*^+*/*+^ or *Grm7*^+*/*−^ controls [31], and other studies have shown that loss of mGlu_7_ only affects short-term synaptic plasticity at SC-CA1 [30]. These findings suggest potential compensation or involvement of an mGlu receptor heterodimer, an emerging theme in mGlu receptor biology [59, 83–86]. Intriguingly, we have recently shown that blockade of LTP induced by mGlu_7_ antagonists or negative allosteric modulators (NAMs) at SC-CA1 may be dependent on an mGlu_7/8_ heterodimer. In these studies, NAMs that blocked both mGlu_7/8_ heterodimers and mGlu_7/7_ homodimers attenuated LTP, whereas NAMs that only blocked mGlu_7/7_ homodimers did not [59]. Our results here could be consistent with a model in which mGlu_7/8_ heterodimer function is involved in LTP under normal conditions (i.e., WT animals), and activity is mediated by an mGlu_8_-containing dimer in *Grm7*^−*/*−^ animals. It is also possible that there is a ceiling effect, and excessive mGlu_7/7_ homodimers are silent in the LTP paradigm used here. Additionally, overexpression of one component of a heterodimer may lead to altered stoichiometry, with potential saturation between different subunits; if this is the case, it does not appear to affect LTP in *GRM7B* overexpressing animals. We are also generating mice that overexpress the *GRM7A* splice variant as a complement to the mouse line reported here, as well as mice in which the mouse Grm_7_ allele is floxed to allow for tissue-specific increases or loss in mGlu_7_ expression, respectively. While differences in activity of the A and B splice variants have not been reported to date, it is possible that effects on LTP may be observed in the *GRM7A* overexpressing line. As the lines are floxed, this will also provide us with a way to systematically compare the effects of the two splice variants in a cell-type specific manner, and these studies are in progress. Additionally, the transgene used in the current studies resulted in a high level of overexpression (roughly fivefold) of the *GRM7B* protein. Titrating protein expression using mouse lines with different levels of expression, viruses to better control overexpression, or small molecules to specifically potentiate mGlu_7_ in a dose-dependent manner will provide additional texture to the consequences of increasing mGlu_7_ expression. Additional future studies could also involve optogenetic or chemogenetic approaches to manipulate specific aspects of neuronal circuitry to localize any effects of mGlu_7_ overexpression. While our previous results suggest an important role for mGlu_7_ in GABAergic neurons [40], neuronal silencing using opsins could provide a mechanism to precisely isolate the effects of mGlu_7_ overexpression.

Our behavioral phenotyping results here show that, in repetitive behavior and cognitive domains, animals overexpressing mGlu_7B_ exhibit abnormalities that mimic those seen in *Grm7*^−/−^ mice [29, 31]. We have not yet explored additional behaviors within these phenotypic domains, which is a limitation of the current work. The observations of abnormal clasping and conditioned fear phenotypes, however, are consistent with reports of patients with duplications of the *GRM7* locus which also result in abnormal phenotypes such as intellectual disabilities (reviewed in [32]). These findings suggest that, like other genes linked to NDDs (i.e., *MECP2*, *TCF4, FRM1*, *CDKL5*, *SHANK3*), mGlu_7_ may have precise dosage requirements in the CNS. In the case of MeCP2, while loss-of-function mutations cause RTT, overexpression of MeCP2 causes the disorder MDS [53]. Despite their opposing molecular origins, patients with RTT and MDS exhibit several overlapping symptoms such as intellectual disability, seizures, and social interaction deficits [53, 87–90]. Intriguingly, the Zoghbi lab has shown that mice modeling RTT and MDS exhibit similar abnormalities in hippocampal circuitry that lead to abnormal, yet similar, synchronous firing [91]. Examination of specific neuronal subtype(s) involved in this process revealed that MeCP2 expression in excitatory neurons is critical to prevent synchrony at baseline, but inhibitory interneurons mediate MeCP2’s ability to prevent strong synchrony when neurons are challenged with factors that affect the balance between excitation and inhibition. Additionally, the authors probed the activity of hippocampal CA1 excitatory projections onto interneurons, which had been proposed to be abnormal in RTT models [92]; these studies showed that spontaneous excitatory postsynaptic currents (sEPSCs) in interneurons in the hippocampal oriens layer were significantly reduced in *Mecp2*^−*/y*^, *Mecp2*^+*/*−^, and *MeCP2-Tg1* (a model of MDS) mice. Impaired responses occurred in mice that lacked MeCP2 in the excitatory projections as well as somatostatin containing inhibitory interneurons (SST-INs), suggesting involvement of both sides of the synapse [91]. As we have found that mGlu_7_ activity may be particularly important in modulating GABA release in area CA1, future studies will examine the consequences of overexpressing and knocking out mGlu_7_ specifically in GABAergic neurons.

It is possible that the reciprocal decrease of mGlu_7A_ may underlie the behavioral changes we have observed in the clasping and conditioned fear studies, and the effect of *GRM7B* overexpression may create a complex knockdown of mGlu_7_. If correct, this could indicate that the excessive levels of *GRM7B* induced in this model may not be active. We would note, however, that the expression of the mouse mGlu_7A_ splice variant in *ON/* + mice was approximately 50% of control levels, suggesting that it might represent a heterozygous condition rather than a knockout. In previous reports, mGlu_7_ heterozygous mice do not exhibit behavioral phenotypes [26, 29, 31]. In contrast, if mGlu_7_ is primarily within a heterodimer and is not functional in vivo as a homodimer (which has been speculated due to the low affinity of mGlu_7_ for glutamate [93]), it is possible that overexpression results in heterodimer saturation. Alternatively, there may be preferential inclusion of mGlu_7A_ versus mGlu_7B_ into functional receptors of either the homodimer or heterodimer form due to differences in their C-termini and their ability to interact with distinct cellular proteins [94–100]. As noted, we are now systematically comparing overexpression of the A and B splice variants to shed light on these possibilities. These studies will involve an in-depth characterization of whole-cell electrophysiology as well as evaluation in additional repetitive, attentional, and cognitive behavioral tasks to move precisely define phenotypes impacted by mGlu_7_ overexpression.

In conclusion, we have generated mice that overexpress the mGlu_7B_ splice variant by approximately fivefold in the brain, resulting in a reciprocal decrease in the mGlu_7A_ splice isoform. These mice exhibit only mild changes in synaptic responses as assessed using field recordings but exhibit abnormal learning and repetitive behavior phenotypes that are similar to *Grm7*^−*/*−^ animals. Overall, under- and over-expressing mGlu_7_ mice provide tools to assist in the understanding of clinical mutations in the *GRM7* gene which lead to severe phenotypes in humans.

## Supplementary Information

Below is the link to the electronic supplementary material.

ESM 1 (PPTX 1.01 MB)

## Data Availability

All data are available by request from the corresponding author.
