# Supplementary material for: Overexpression of mGlu7B in Mice: Implications for Neurodevelopmental Disorders
Source: Mol Neurobiol. 2025 Jul 10;62(11):14015–31. doi: 10.1007/s12035-025-05183-y (PMC12511187; doi:10.1007/s12035-025-05183-y)
Supplement: Supplementary file 1 — (PPTX 1.01 MB) [file 12035_2025_5183_MOESM1_ESM.pptx]

## Slide 1
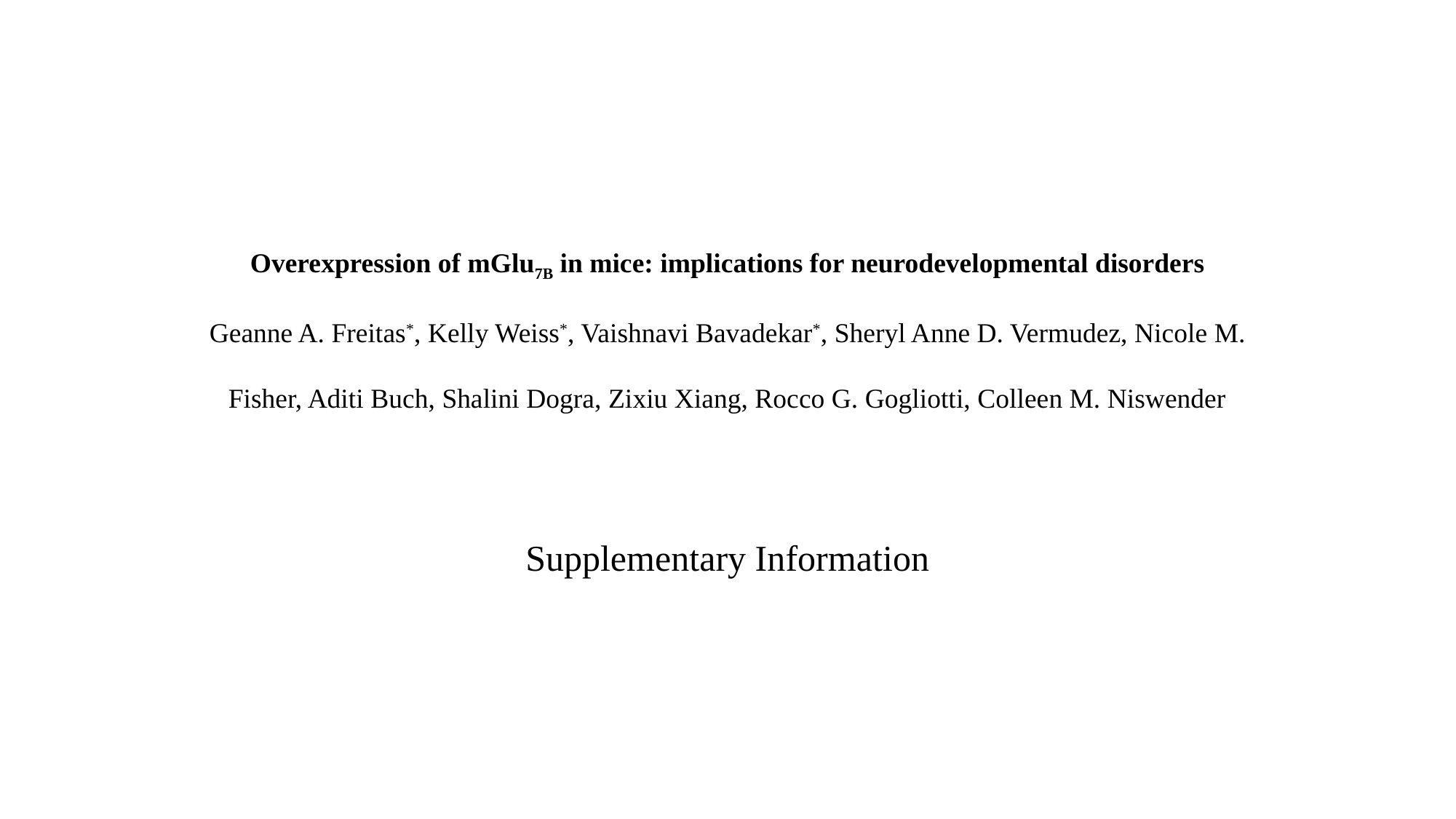

# Overexpression of mGlu7B in mice: implications for neurodevelopmental disorders Geanne A. Freitas*, Kelly Weiss*, Vaishnavi Bavadekar*, Sheryl Anne D. Vermudez, Nicole M. Fisher, Aditi Buch, Shalini Dogra, Zixiu Xiang, Rocco G. Gogliotti, Colleen M. Niswender
Supplementary Information

## Slide 2
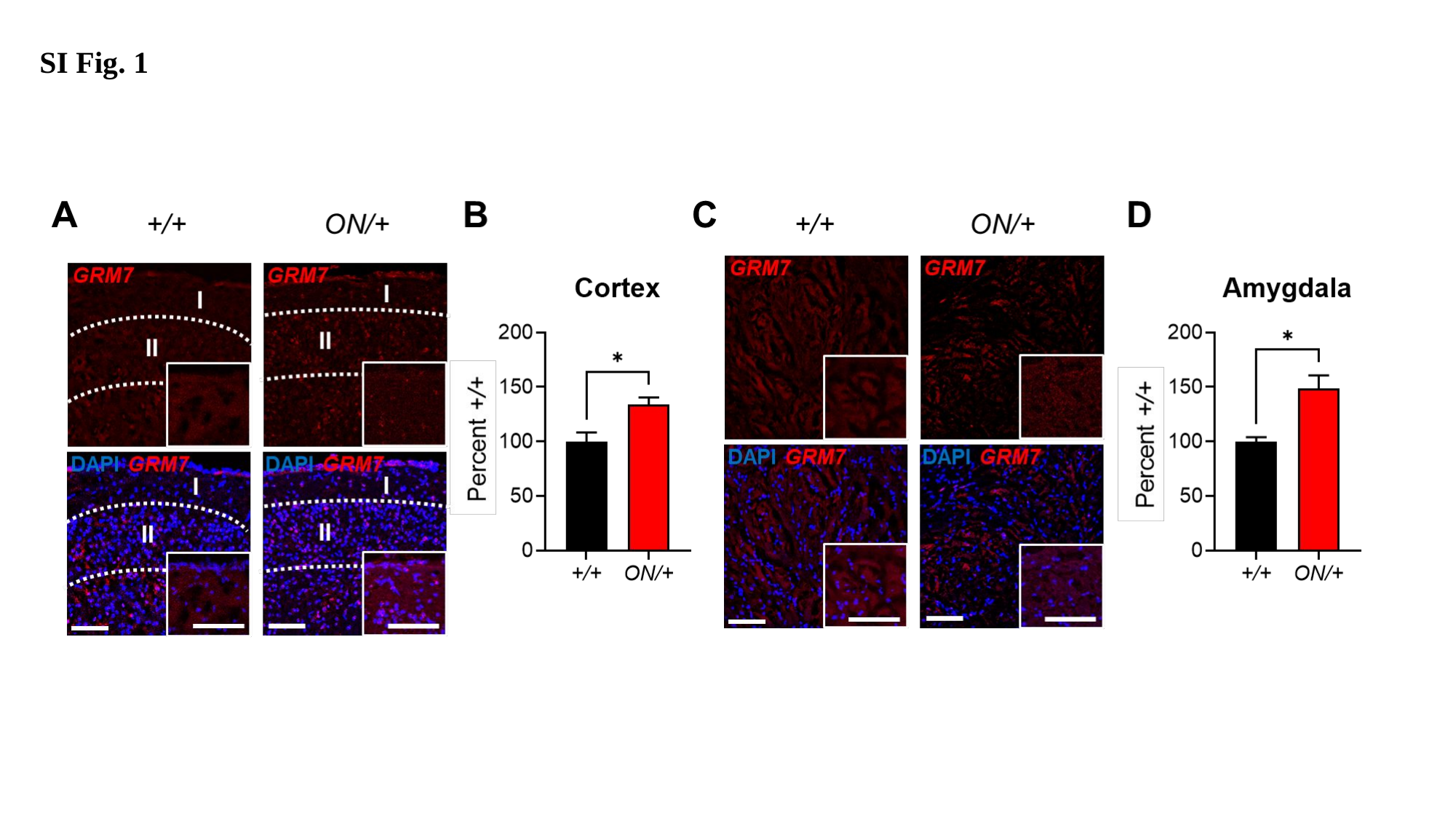

SI Fig. 1

## Slide 3
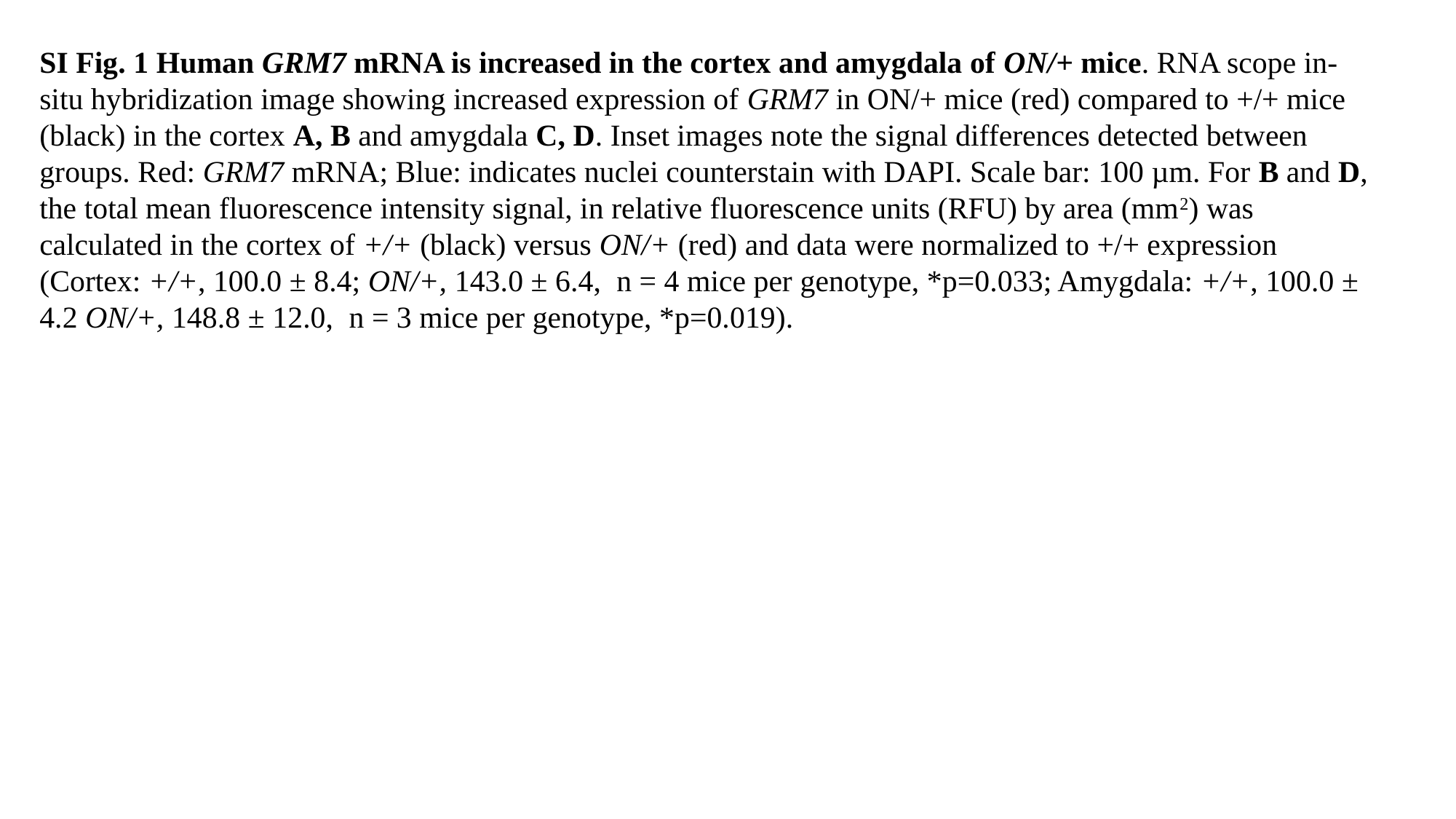

SI Fig. 1 Human GRM7 mRNA is increased in the cortex and amygdala of ON/+ mice. RNA scope in-situ hybridization image showing increased expression of GRM7 in ON/+ mice (red) compared to +/+ mice (black) in the cortex A, B and amygdala C, D. Inset images note the signal differences detected between groups. Red: GRM7 mRNA; Blue: indicates nuclei counterstain with DAPI. Scale bar: 100 µm. For B and D, the total mean fluorescence intensity signal, in relative fluorescence units (RFU) by area (mm2) was calculated in the cortex of +/+ (black) versus ON/+ (red) and data were normalized to +/+ expression (Cortex: +/+, 100.0 ± 8.4; ON/+, 143.0 ± 6.4, n = 4 mice per genotype, *p=0.033; Amygdala: +/+, 100.0 ± 4.2 ON/+, 148.8 ± 12.0, n = 3 mice per genotype, *p=0.019).

## Slide 4
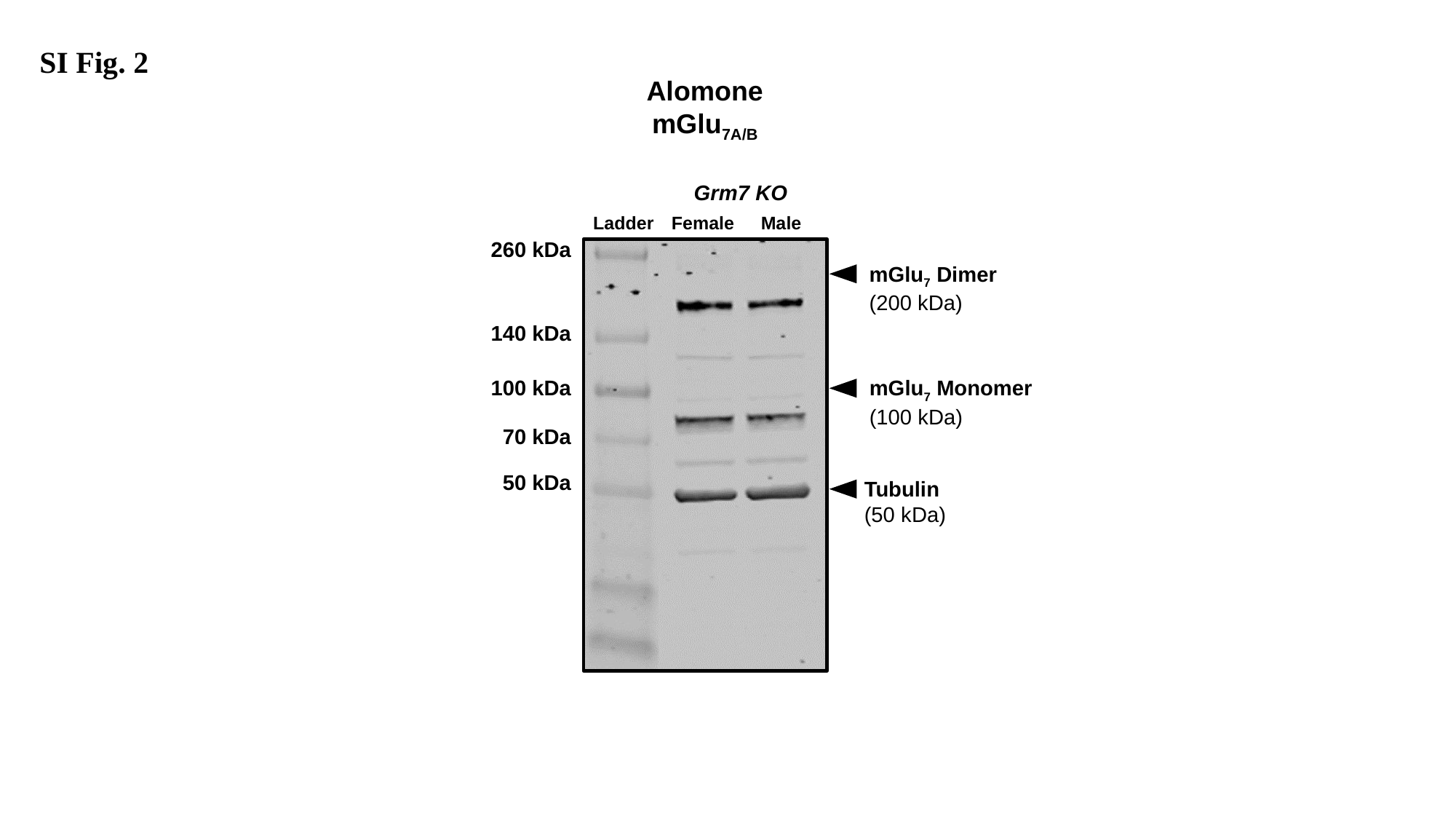

SI Fig. 2
Alomone
mGlu7A/B
Grm7 KO
Ladder
Female
Male
260 kDa
mGlu7 Dimer
(200 kDa)
140 kDa
100 kDa
mGlu7 Monomer
(100 kDa)
70 kDa
50 kDa
Tubulin
(50 kDa)

## Slide 5
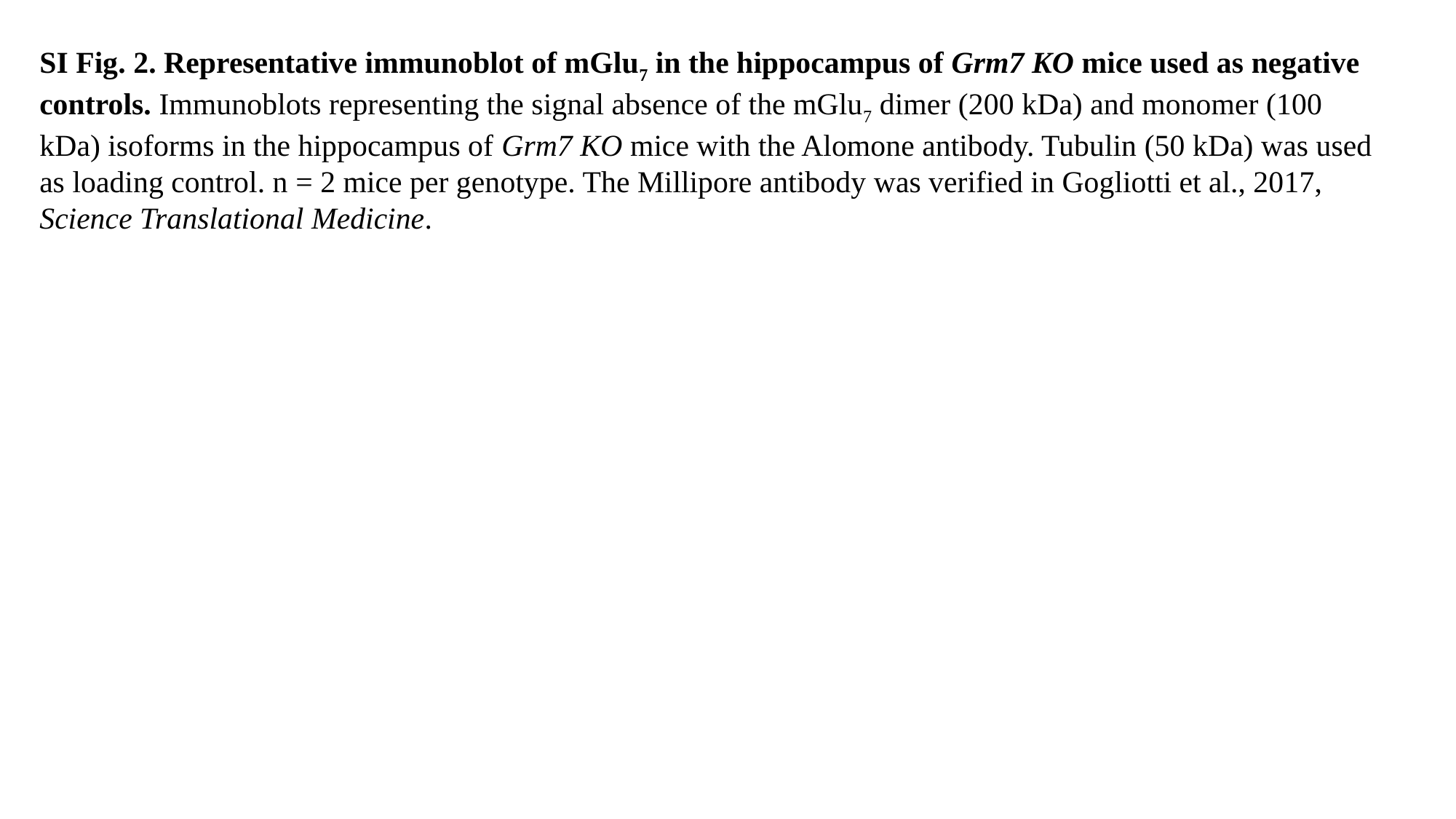

SI Fig. 2. Representative immunoblot of mGlu7 in the hippocampus of Grm7 KO mice used as negative controls. Immunoblots representing the signal absence of the mGlu7 dimer (200 kDa) and monomer (100 kDa) isoforms in the hippocampus of Grm7 KO mice with the Alomone antibody. Tubulin (50 kDa) was used as loading control. n = 2 mice per genotype. The Millipore antibody was verified in Gogliotti et al., 2017, Science Translational Medicine.
